# Memory CD4 T cell-derived IL-2 synergizes with viral infection to exacerbate lung inflammation

**DOI:** 10.1371/journal.ppat.1007989

**Published:** 2019-08-14

**Authors:** K. Kai McKinstry, Fahmida Alam, Valeria Flores-Malavet, Mate Z. Nagy, Stewart Sell, Andrea M. Cooper, Susan L. Swain, Tara M. Strutt

**Affiliations:** 1 Immunity and Pathogenesis Division, Burnett School of Biomedical Sciences, College of Medicine, University of Central Florida, Orlando, Florida, United States of America; 2 Department of Health, Wadsworth Center, Albany, New York, United States of America; 3 Trudeau Institute, Saranac Lake, New York, United States of America; 4 Department of Pathology, University of Massachusetts Medical School, Worcester, Massachusetts, United States of America; University of Rochester Medical Center, UNITED STATES

## Abstract

Defining the most penetrating correlates of protective memory T cells is key for designing improved vaccines and T cell therapies. Here, we evaluate how interleukin (IL-2) production by memory CD4 T cells, a widely held indicator of their protective potential, impacts immune responses against murine influenza A virus (IAV). Unexpectedly, we show that IL-2-deficient memory CD4 T cells are more effective on a per cell basis at combating IAV than wild-type memory cells that produce IL-2. Improved outcomes orchestrated by IL-2-deficient cells include reduced weight loss and improved respiratory function that correlate with reduced levels of a broad array of inflammatory factors in the infected lung. Blocking CD70-CD27 signals to reduce CD4 T cell IL-2 production tempers the inflammation induced by wild-type memory CD4 T cells and improves the outcome of IAV infection in vaccinated mice. Finally, we show that IL-2 administration drives rapid and extremely potent lung inflammation involving NK cells, which can synergize with sublethal IAV infection to promote acute death. These results suggest that IL-2 production is not necessarily an indicator of protective CD4 T cells, and that the lung environment is particularly sensitive to IL-2-induced inflammation during viral infection.

## Author summary

We show that memory CD4 T cell mediated protection against influenza A virus is independent of the signature multifunctional cytokine IL-2 that is thought to define the most protective memory cells. IL-2 deficient cells are more effective than wild-type memory cells on a per cell basis at combating IAV and drive tempered early innate inflammatory responses. Our studies define a clear and surprising role for IL-2 as a cytokine adjuvant within the lung that can synergize with virus driven acute inflammatory responses to cause morbidity during sublethal respiratory viral infection.

## Introduction

Interleukin-2 (IL-2) produced by CD4 T cells is thought to be critical for orchestrating optimal immune responses by acting as an autocrine growth and survival factor [1] as well as a paracrine cytokine to enhance the activity of other cell types, notably NK cells and CD8 T cells [2, 3]. IL-2 production by T cells is strictly regulated by antigen recognition and costimulatory signals, resulting in its transient secretion during cognate interactions with activated APC [4]. A distinguishing feature of resting memory and memory-derived secondary CD4 T cell effectors is their ability to produce higher levels of IL-2 more rapidly than naïve and primary CD4 T effector cells [5, 6] and CD8 T cells [7, 8]. Consequently, memory CD4 T cells are the most physiologically relevant source of IL-2 *in vivo*.

The capacity of Th1-polarized memory cells to co-produce high levels of IL-2 in combination with IFN-γ is widely held as a marker of superior protective capacity [9]. Indeed, memory CD4 T cells marked by dual production of IFN-γ and IL-2 provide robust immunity against influenza A virus (IAV) challenge in murine models [10–12]. Large numbers of memory cells capable of producing IL-2 and responding rapidly against IAV have also been characterized in human lungs [13, 14] and transcriptional signatures, phenotypes, and functional analysis support that lung-resident memory CD4 T cells are strong producers of IL-2 [12, 15]. Importantly, the presence of increased numbers of IAV-specific memory CD4 T cells prior to seasonal infection correlates with improved clinical outcome in human longitudinal studies [16]. Conversely, severe influenza disease has been associated with decreased levels of IL-2 in the lung [17]. These findings support the concept that IL-2 production is essential for optimizing immunity against IAV mediated by memory CD4 T cells. Here, we directly test this hypothesis.

To overcome major technical and physiological barriers that prevent the straight forward investigation of the impact of memory CD4 T cell-derived IL-2 *in vivo*, we generated memory CD4 T cells specific for IAV *in vitro* from naïve TcR transgenic precursors in the presence of exogenous IL-2 [5]. This model provides IL-2-dependent signals to IL-2-deficient (*Il2*^*-/-*^) CD4 T cells during priming that are essential to form most CD4 memory populations by programing IL-7 receptor expression and rescuing effector cells from apoptosis [18, 19]. We used WT and *Il2*^-/-^ memory cells generated in this way in a well-established adoptive transfer model in which naive host mice are challenged with IAV recognized by the donor cells. This reductionist approach overcomes complications associated with long-term blockade of IL-2 or IL-2 receptors by antibody administration which has off target effects through the disruption of FoxP3^+^ T regulatory (T reg) function that can independently impact the outcome of infections, including secondary IAV challenge [20]. Additionally, while conditional knock-out models that rely on the targeted expression of cre-recombinase to delete floxed genes are widely employed, the efficiency of inducible knock systems dependent upon tamoxifen-induced cre-recombinase expression can have varied efficiency within different tissues that can confound observations [21].

Unexpectedly, we find that *Il2*^*-/-*^ IAV-specific memory CD4 T cells provide superior protection against IAV compared to WT memory cells of the same specificity. Improved outcomes associated with *Il2*^*-/-*^ responses include wide-ranging reductions in the constituents of pulmonary and systemic inflammation, accelerated viral clearance, improved pulmonary mechanics, and reduced weight loss. We also show that blocking the CD70-CD27 costimulatory pathway to dramatically reduce IL-2 production by memory CD4 T cells [19] tempers inflammation and morbidity in an adoptive transfer model as well as in intact vaccinated wildtype mice. Finally, we show that IL-2 administration to naive mice directly and rapidly upregulates a broad array of cytokines and chemokines in the lung and synergistically enhances inflammation induced by IAV, indicating that the lung is particularly sensitive to IL-2 driven-inflammation. NK cells are major contributors to pulmonary IL-2-induced inflammation. Moreover, NK cell depletion during IAV challenge of recipients of WT memory CD4 T cells phenocopies the improved outcomes seen in *Il2*^*-/-*^ memory CD4 T cell recipients.

Overall, our results indicate that memory CD4 T cell-derived IL-2 acts as a potent adjuvant in the lung that enhances the production of a broad early inflammatory response during acute viral infection. We find that IL-2 synergizes with pathogen-driven stimulation of innate immunity to drive robust inflammatory responses that can worsen outcomes during primary IAV infection as well as in models of memory CD4 T cell-mediated protection against lethal IAV challenge. Our findings have important implications for therapies aimed at reducing the severity of IAV infection, for the correlates of protection used to design and evaluate T cell-based vaccines, and for the consequences of respiratory infection during the clinical use of therapeutics that result in increased levels of IL-2. This work may also help provide mechanistic insight into the immunopathological impact of memory CD4 T cells induced by vaccination in models of chronic infection [22, 23].

## Results

### Increased levels of IL-2 are detected during heterosubtypic challenge of IAV-primed mice

**Fig 1:**
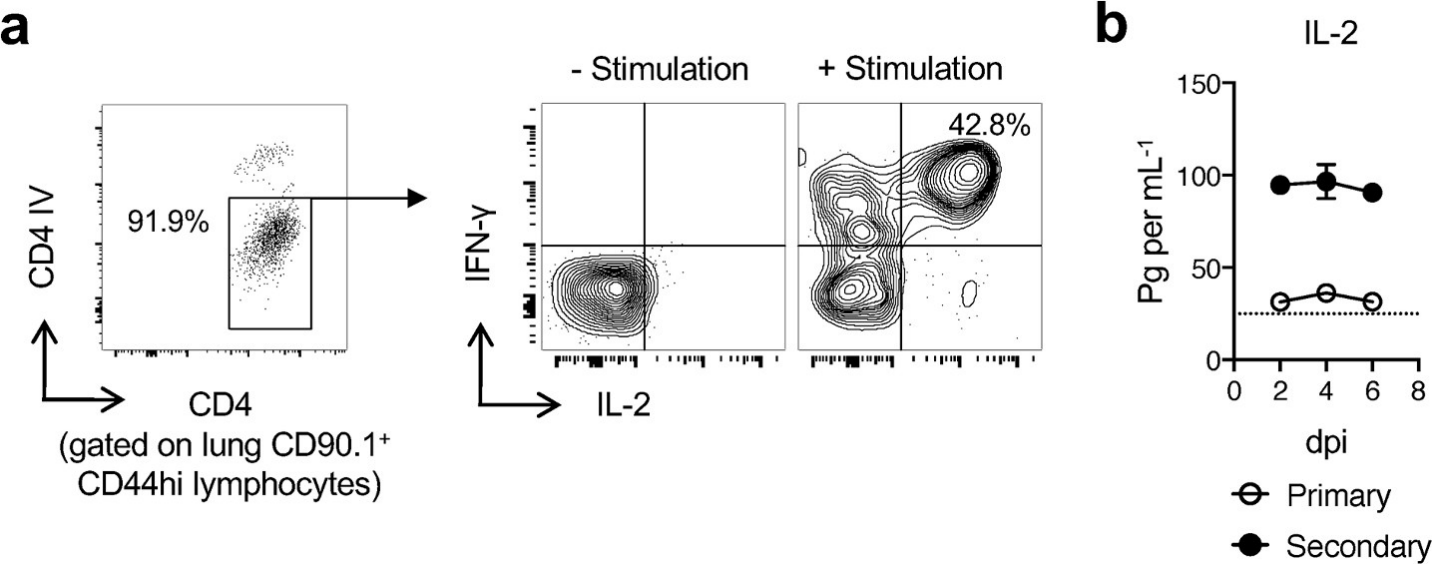
Increased levels of IL-2 are detected during heterosubtypic challenge of IAV-primed mice. IAV-primed BALB/c were infected with 0.2 LD_50_ A/PR8 virus and IFN-γ and IL-2 production by CD44hi CD4 T_RM_ analyzed at 65 dpi by ICCS following stimulation with PMA and ionomycin (**a**). Levels of IL-2 in lung homogenates on the indicated days post 10 LD_50_ heterosubtypic A/PR8 virus challenge of unprimed and animals primed 35 days previously with cold-adapted A/Alaska (**b**) (3–5 mice per group per day; 1 of 2 experiments). All error bars represent the standard deviation.

We previously found that IAV-specific lung-resident memory CD4 T cells generated from naive TcR Tg donor cells displayed robust IL-2 production when assayed at day 30 post-infection and beyond [12]. We used shielding from labeling by i.v. administered anti-CD4 Ab to discriminate lung-resident from circulating CD44hi CD4 T cells in intact IAV-primed mice and found that the shielded cells displayed strong IL-2 production via intracellular cytokine staining following stimulation with PMA and ionomycin (**Fig 1A**). This confirms that endogenous, polyclonal lung-resident memory CD4 T cells display a strong capacity to produce IL-2. We thus reasoned that IL-2 signals may have a greater impact on immune responses against heterosubtypic IAV infection than during primary responses against IAV where IL-2-producing CD4 T cells only reach the lung in significant numbers at 6 or 7 days post-infection [24]. Indeed, we detected significant levels of IL-2 in the lungs of mice during the first week of heterosubtypic IAV infection while IL-2 was barely detectable during the same timeframe of a primary IAV response (**Fig 1B**).

In order to access the role of IL-2 production by memory CD4 T cells during their antigenic recall, we used a previously validated model employing WT and *Il2*^*-/-*^ TcR transgenic CD4 T cells to generate memory populations from Th1-polarized effector populations *in vitro* [5]. Importantly, when transferred to naïve adoptive hosts that are then challenged with IAV, these memory cell responses mirror key elements of the endogenous CD4 T cell recall response against IAV [8, 10, 19]. Briefly, we provided exogenous IL-2 to cultures of naïve WT or *Il2*^*-/-*^ DO11.10 TcR transgenic cells [25] to program their capacity to form memory [19]. The resulting effectors cells were cultured *in vitro* in the absence of antigen and inflammatory signals for 3 days during which they transition to a resting state virtually indistinguishable from long-term memory CD4 T cells generated *in vivo* [5, 19]. We have used such *in vitro*-generated memory cells in adoptive transfer studies to determine key mechanisms of CD4 T cell-mediated protection against IAV [26, 27].

To clearly delineate protective functions of memory CD4 T cells versus those provided by memory CD8 T cells, memory B cells, and other primed populations that would not be feasible to block in intact IAV-primed mice [26], we transferred an equal number of WT or *Il2*^*-/-*^ DO11.10 memory cells to unprimed mice then infected with A/PR8-OVA_II_ that contains the OVA_323-339_ epitope recognized by the DO11.10 TcR. We gave 5x10^6^ memory cells, which results in ~5x10^5^ cells able to respond factoring in a ‘10% take’ [28]. As previously discussed [26], this number is in the range of the estimate of the total number of memory CD4 T cells generated by IAV priming in BALB/c mice, as well as in studies analyzing DR-1 “humanized” transgenic mice [29] in which the magnitude of the total HA-specific memory CD4^+^ T cell response detected by ELISPOT assay alone is about 1x10^5^ cells. Given that not all cells are expected to make the cytokines assayed in the ELISPOT, and assuming that the response against HA accounts for 20–50% of the total IAV specific cells [29, 30], a conservative estimate of the total memory CD4 T cell pool is in the range of 2-5x10^5^. The recipient mice were challenged with a lethal (2 LD_50_) dose of IAV against which 5x10^6^ WT memory CD4 T cells protects [26] in order to test as stringently as possible the role of IL-2 production by the memory CD4 T cells during a protective response.

### Early memory CD4 T cell-enhanced inflammation is tempered in the absence of IL-2

**Fig 2:**
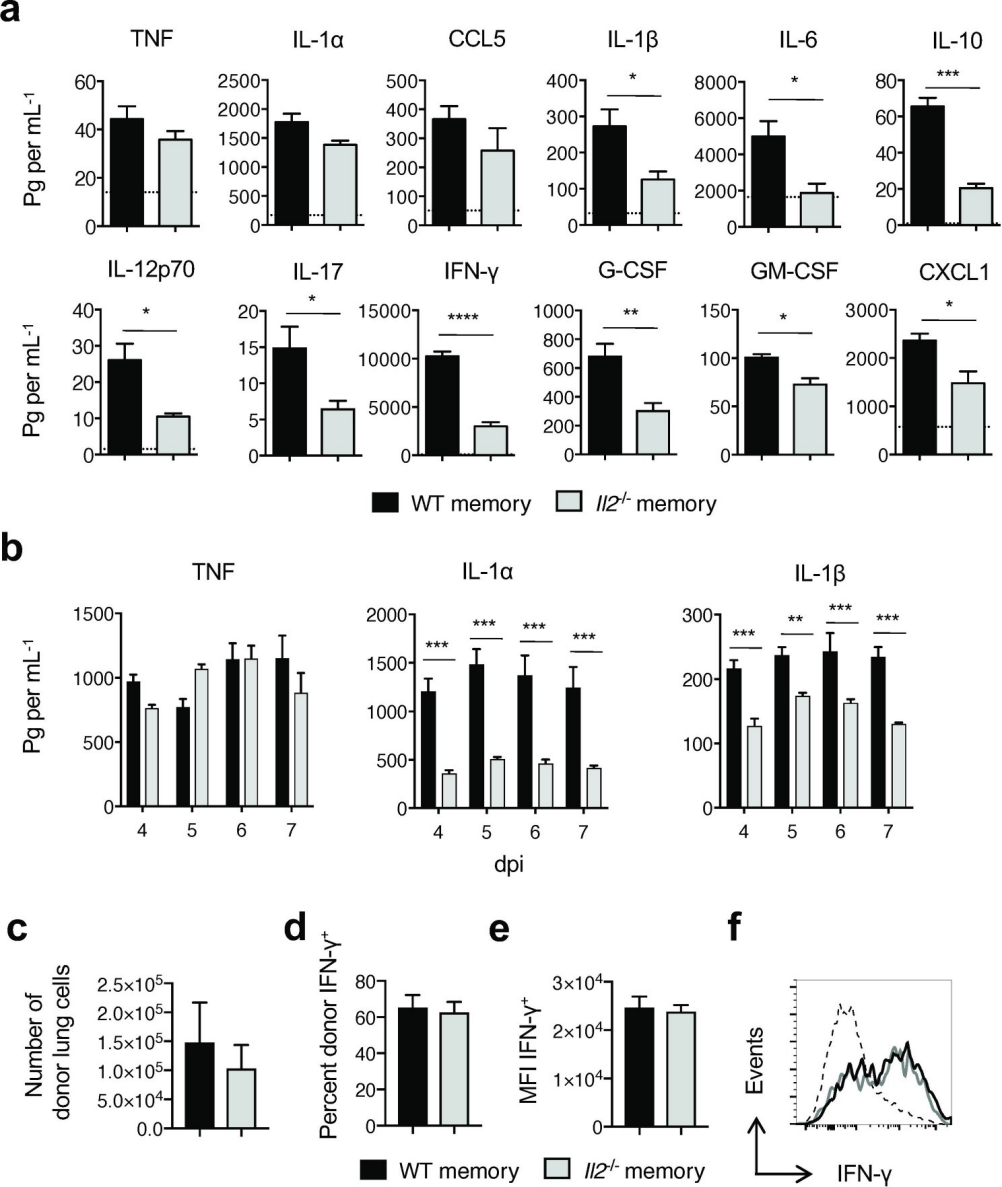
*IL2*^*-/-*^ memory CD4 cells induce less inflammation than WT memory cells. Unprimed BALB/c recipients of WT or *Il2*^*-/-*^ memory DO11.10 CD4 T cells were challenged with a 2 LD_50_ dose of A/PR8-OVA_II_ virus. On day 3, inflammatory responses in lung homogenates were measured (**a**) (3 mice per group; 1 of 3 experiments). (**b**) The amount of TNF, IL-1α, and IL-1β detected kinetically through 7 dpi (3 mice per group; 1 of 2 experiments). The number of donor cells within the lung of recipients of WT or *Il2*^*-/-*^ memory DO11.10 CD4 T cells (**c**), and the frequency and MFI of donor IFN-γ production were also determined (**d**-**f**). In (a) the dashed line represents analyte levels detected on 3dpi in the absence of memory CD4 T cell adoptive transfer and in (f) the dashed line represents IFN-γ production from unstimulated controls. All error bars represent the standard deviation, and * *P* < 0.05, ** *P* < 0.01, *** *P* < 0.001, **** *P* < 0.0001.

The earliest protective function of memory CD4 T cells upon cognate recognition of antigen during IAV challenge is to ‘jump-start’ innate inflammatory responses in the lung. This innate inflammatory response leads to marked control of viral titers within 3 days of infection (dpi), is generated independently of Type I and II IFN, TNF, and pathogen associated molecular pattern (PAMP) recognition [31], and is likely driven by resident memory T (T_RM_) cells. To analyze the role of IL-2 production by memory CD4 T cells in promoting this induction of innate immunity, we assessed pulmonary inflammation in naïve recipients of WT or *Il2*^*-/-*^ memory CD4 T cells at 3 dpi with A/PR8-OVA_II_. As expected from previous studies [31], WT memory CD4 T cells induced significantly higher levels of a broad spectrum of inflammatory cytokines and chemokines by 3 dpi compared to control mice not receiving memory cells (represented as dashed lines in graphs) (**Fig 2**). With the exception of TNF, IL-1α, and CCL5 that were detected at levels comparable to those in hosts with WT memory CD4 T cells, recipients of *Il2*^-/-^ memory cells displayed markedly reduced levels of the factors analyzed (**Fig 2A**), the majority of which were nevertheless significantly higher than in control mice. To evaluate how long the inflammatory factors remained reduced in the *Il2*^*-/-*^ versus WT memory CD4 T cell recipients, we examined lung levels from 4–7 dpi. TNF, which is itself produced by WT and *Il2*^-/-^ memory CD4 T cells responding to IAV [19], remained similar. In contrast, IL-1α and IL-1β were reduced when assessed at day 4 and remained lower through 7 dpi in recipients of *Il2*^*-/-*^ memory CD4 T cells (**Fig 2B**). As no differences are seen in donor memory CD4 T cell numbers or in IFN-γ production at d4 and d7 (**Fig 2C–2F**), these results suggest that IL-2 production from memory CD4 T cells promotes potent, acute, and broad production of inflammatory factors in the lung of naïve recipient mice. The kinetic timeframe of the memory CD4 T cell-driven enhanced lungs of recipient mice inflammatory response observed here mirrors previous findings and is in line with the response seen in IAV-primed mice post-heterosubtypic challenge [31].

### IL-2 from memory CD4 T cells is not required for protection against IAV

**Fig 3:**
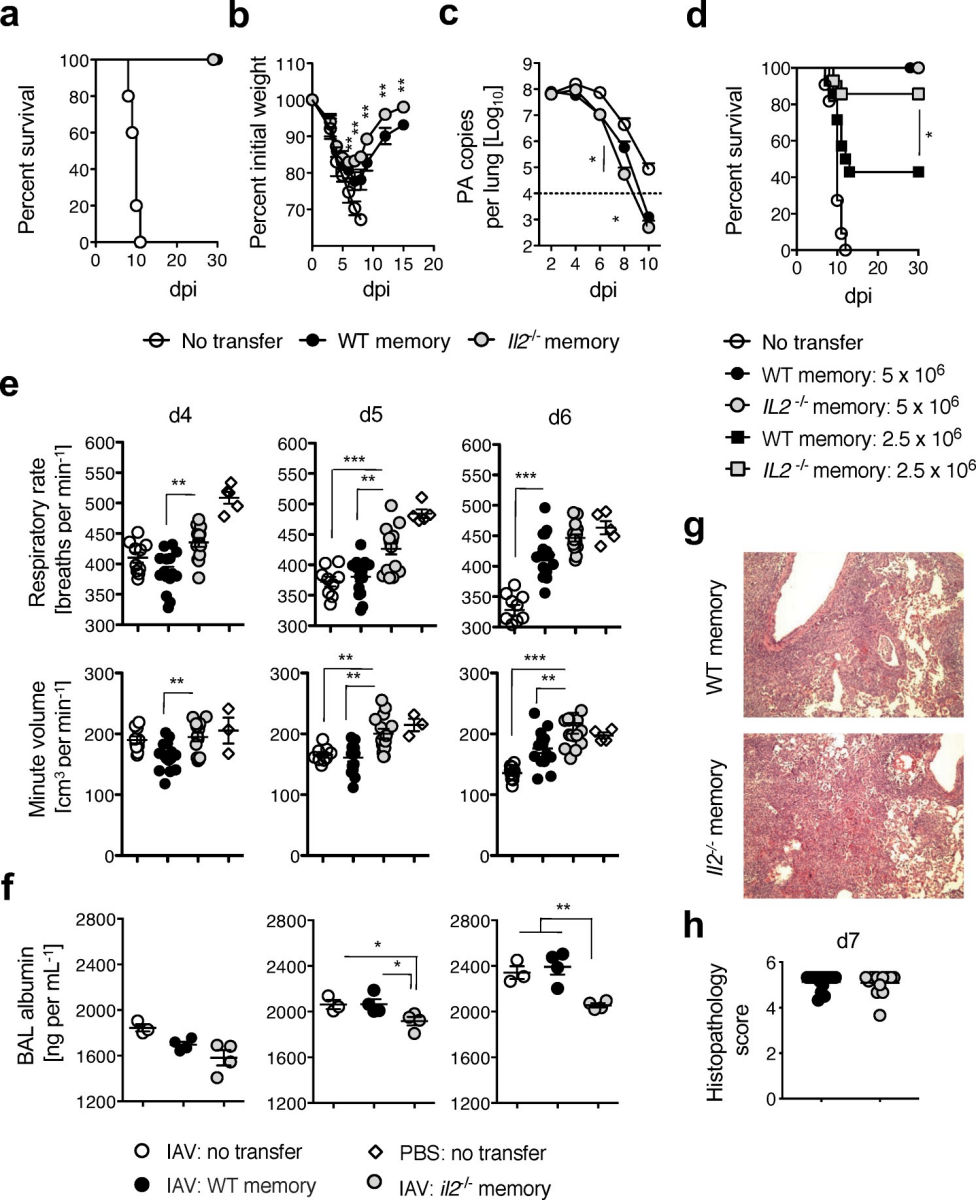
IL-2 production is not needed for IAV-specific memory CD4 T cell-mediated protection. Unprimed BALB/c hosts received WT or *Il2*^*-/-*^ memory DO11.10 CD4 T cells and were challenged with a 2 LD_50_ dose of A/PR8-OVA_II_ virus. Survival (**a**), morbidity (**b**), and viral titer (**c**) were assessed (3–5 mice per group per day; 1 of 3 experiments). In separate experiments, the number of adoptively transferred memory CD4 T cells was varied as indicated and (**d**) survival during lethal infection monitored (5 mice per group, 1 of 3 experiments). On the indicated days, respiratory rate, minute volume (**e**), BAL albumin levels (**f**), as well as histopathology on 7 dpi (**g** and **h**) were assessed, representative images of lungs at 40X are shown (3–15 mice per group per day; 1 of 2 experiments). All error bars represent the standard deviation and * *P* < 0.05, ** *P* < 0.01, *** *P* < 0.001).

The changes seen above could impact the ability of WT versus *Il2*^*-/-*^ memory CD4 T cells to protect against infection. To evaluate this, we assessed a number of parameters associated with recovery from IAV challenge including morbidity, mortality, and viral clearance. All naïve recipients of memory CD4 T cells survived, while control mice not receiving memory cells succumbed by 10 dpi (**Fig 3A**). Strikingly though, recipients of *Il2*^*-/-*^ memory CD4 T cells began to recover weight 2–3 days earlier than WT recipients **(Fig 3B)**. The improved kinetics of weight recovery in *Il2*^*-/-*^ memory CD4 T cell recipients correlated with modest but significantly accelerated viral clearance at 8 and 10 dpi **(Fig 3C).** When the number of cells transferred was titrated, the enhanced protective capacity of *Il2*^*-/-*^ memory CD4 T cells was even more evident (**Fig 3D**), supporting the concept that reduced inflammatory responses driven by the memory CD4 T cells in the absence of IL-2 signaling correlate with improved outcomes.

As in previous studies [19], we found no differences between the frequency and proliferation of WT and *Il2*^*-/-*^ memory CD4 T cells in the spleen, draining lymph nodes, and lungs on 4 and 7 dpi (**S1 Fig**), indicating that the enhanced protective capacity of *Il2*^*-/-*^ memory CD4 T cells and differences in the inflammatory response observed are not due to differences in the kinetics or peak magnitude of WT versus *Il2*^*-/-*^ memory CD4 T cell responses. Similar patterns of improved recovery in recipients of *Il2*^*-/-*^ memory cells were seen in nude hosts lacking T cells, J_H_D hosts lacking B cells, and SCID hosts deficient in both T and B cells **(S2 Fig).** These observations argue against the possibility that altered helper functions impacting anti-viral B cells or CD8 T cell responses, or altered activity of IL-2 dependent host regulatory T cells impact the differences seen.

We and others previously found significant differences in the pulmonary function of unprotected versus protected animals during the first week of IAV challenge [32–34]. We thus analyzed respiratory mechanics in naïve recipients of WT or *Il2*^-/-^ memory CD4 T cell and found that recipients of *Il2*^*-/-*^ cells demonstrated improved respiratory rates and pulmonary minute volumes from 4 to 5 dpi. On 6 dpi, both groups of memory CD4 T cells recipients began to show signs of recovery in respiratory rates and only minute volumes remained significantly different **(Fig 3E)**. Furthermore, lower levels of serum albumin, a measure of vascular leak, were detected in the bronchoalveolar lavage of *Il2*^*-/-*^ versus WT memory CD4 T cell recipients on 5 and 6 dpi **(Fig 3F)**, indicating reduced pulmonary edema. Interestingly, histopathologic analysis of the lung did not reveal marked differences between recipients of WT or *Il2*^*-/-*^ cells at 7 dpi **(Fig 3G and 3H)**. Together, these results indicate that early IL-2 production by memory CD4 T cells responding to IAV amplifies inflammatory responses that impair pulmonary function and promote pulmonary edema without causing measurable increases in immunopathology.

### Blocking CD70-mediated signaling tempers memory CD4 T cell-dependent inflammation

**Fig 4:**
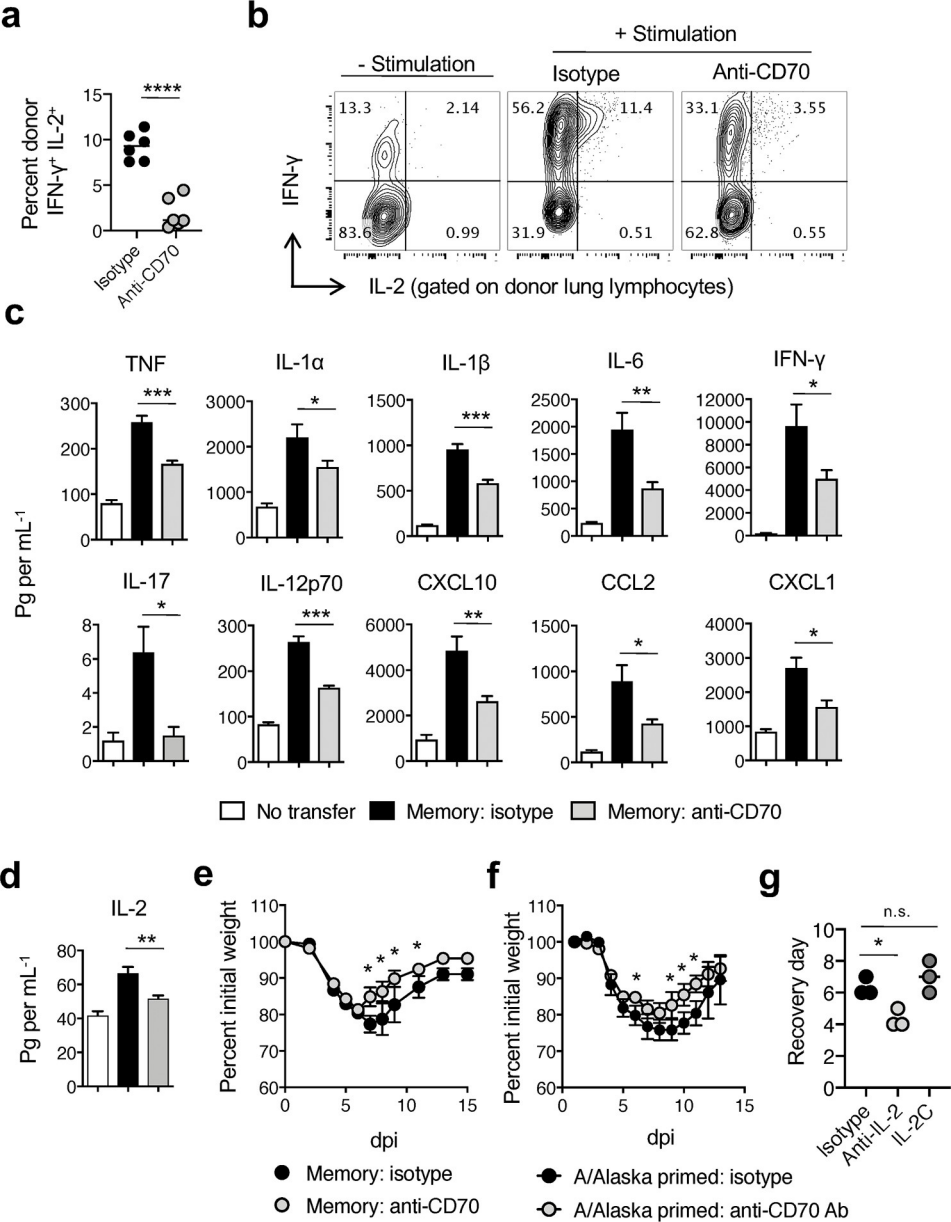
Blocking CD70-mediated signaling tempers early memory CD4 T cell-driven inflammation during IAV. Unprimed BALB/c recipients of memory WT DO11.10 CD4 T cells were infected with a 2 LD_50_ dose of A/PR8-OVA_II_ virus and treated with isotype or anti-CD70 blocking antibody (Ab). On 7 dpi, IFN-γ and IL-2 production by donor cells in the lungs was determined by ICCS following peptide stimulation (**a**). Representative cytokine production is shown in (**b**). On day 3, inflammatory responses (**c**) and levels of IL-2 (**d**) in lung homogenates were measured and are presented (3 mice per group; 1 of 3 experiments). In separate experiments, morbidity (**e**) was assessed in recipients of WT memory CD4 T cells as well as (**f**) following 10 LD_50_ heterosubtypic virus recall challenge of CD8 depleted, cold-adapted A/Alaska-primed animals treated with anti-CD70. In separate groups of animals treated as in (f), anti-IL-2 neutralizing Ab or IL-2C containing 2 μg of IL-2 was administered i.p. for 4 days and recovery from morbidity monitored (**g**) (3–4 mice per group; 1 of 2 experiments). All error bars represent the standard deviation, and * *P* < 0.05, ** *P* < 0.01, *** *P* < 0.001, and **** *P* < 0.0001.

Given that memory CD4 T cell derived-IL-2 appears to amplify IAV-associated inflammatory responses, modulating IL-2 production may serve as a therapeutic strategy to decrease morbidity. Treating mice with a blocking antibody against CD70 significantly reduces IL-2 and IFN-γ production from memory CD4 T cells responding in the lung at 7 dpi with IAV (**Fig 4A** and **4B**) but does not impact their response kinetics or ability to control virus [19]. Similar control of IL-2 production from CD4 and CD8 T cells by CD70-dependent signals has been found in other infection models [35, 36]. We thus asked if blocking CD70 as a means to reduce IL-2 production could improve the outcome of WT memory CD4 T cell-mediated protection. Much like the recipients of *Il2*^-/-^ memory CD4 T cells in Fig 2, recipients of WT memory CD4 T cells treated with anti-CD70 antibody showed reduced levels of IL-1α, IL-1β, IFN-γ, IL-6, IL-17, CCL2 (MCP-1), CXCL1 (KC), and IL-12 (**Fig 4C**), many of which are associated with exacerbated IAV infection [37–40]. In contrast to these observations, we previously found memory CD4 T cell-induced levels of IL-1, IL-6, CCL2, CXCL1, and IL-12 to be similar in WT and IFN-γ-receptor knockout mice following IAV challenge [31], arguing against changes in IFN-γ production by memory CD4 T cells following anti-CD70 antibody treatment contributing to the patterns seen in Fig 4C. Instead, the significantly lower amount of IL-2 in lung homogenates with CD70 blockade (**Fig 4D**), supports the position that a reduction in the amount of IL-2 available for paracrine signaling plays a major role in the tempered inflammatory responses observed. The reduced inflammatory response and corresponding small but significant reduction in weight loss and faster recovery (**Fig 4E**) seen with CD70 blockade largely phenocopies observations with *Il2*^*-/-*^ memory CD4 T cell transfer in Figs 2 and 3.

To test whether blocking CD70 to reduce memory CD4 T cell-driven inflammation could improve outcomes in a more translational setting, naïve WT mice were primed with a cold-adapted vaccine strain of IAV (A/Alaska; H2N2) and were challenged after 35 days with A/PR8 (H1N1). Groups of primed mice were treated with CD70 blocking antibody or an isotype control only during heterosubtypic A/PR8 infection. Given that memory CD8 T cell responses are largely independent of CD27:CD70 signaling [41] and since they can provide strong protection independently of the CD4 T cell recall response [42], to specifically address the isolated impact of CD4 T cell mediated protection IAV-primed mice were also depleted of CD8 T cells prior to heterosubtypic challenge. All primed mice survived and CD70 blockade improved the time to recovery of the mice by 2–3 days (**Fig 4F**). In contrast to CD70 blockade as well as treatment with IL-2 neutralizing antibodies, administration of exogenous IL-2 to mice challenged with IAV failed to improve recovery (**Fig 4G**). These findings suggest that the protective efficacy of vaccine-primed memory CD4 T cells can be improved by reducing their IL-2 production.

### IL-2 directly drives broad lung inflammation that synergizes with viral infection

Our results imply that the improved protective efficacy of the *Il2*^*-/-*^ memory CD4 T cells in the adoptive transfer model employed here is due largely to differences in the inflammatory response generated upon infection. IL-2 is known to activate innate and adaptive immune cells such as NK cells and CD8 T cells but also drives T reg responses that have anti-inflammatory actions [43]. Though administration of IL-2 has been reported to promote systemic inflammation and febrile illness in cancer patients [44], detailed analysis of how IL-2 impacts the lung environment is lacking. We thus analyzed whether providing IL-2 in the absence of memory CD4 T cell transfer would directly enhance inflammatory cytokine and chemokine expression in the lung, even in the absence of IAV infection.

We first administered soluble IL-2 or IL-2:anti-IL-2 antibody (clone S4B6) complexes (IL-2C) [45–47] to unmanipulated mice by i.p. injection for 3 consecutive days and analyzed lung homogenates and serum for changes in cytokine and chemokine expression. We previously found that this regime, employing 2 μg of IL-2, restored memory CD4 T cell generation from *Il2*^*-/-*^ CD4 T cells to WT levels in an IAV model, indicating its ability to deliver physiologically relevant IL-2 signals *in vivo* [19]. Strikingly, IL-2C treatment drove strong expression of a number of analytes such as IL-6, IFN-γ, IL-17, and G-CSF (**S3A Fig**) detected in Fig 2, particularly in the lung and to a lesser extent in the serum.

**Fig 5:**
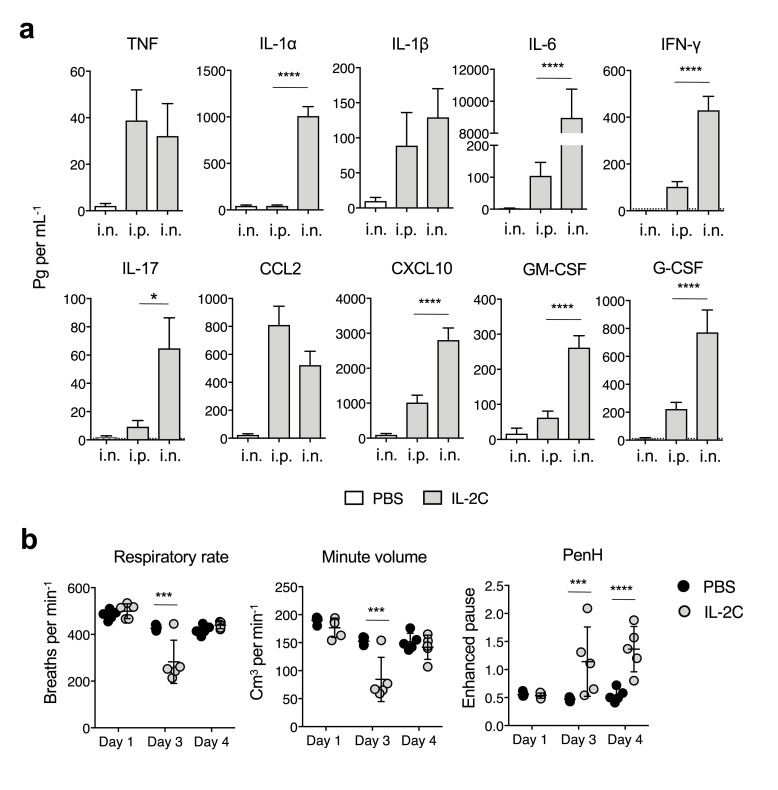
IL-2 drives potent inflammatory responses in the lung that impair respiratory mechanics. IL-2Cs containing 2 μg of IL-2 were administered to naive BALB/c mice either i.p. or i.n. for 3 days. On d4, (**a**) inflammatory responses in lung homogenates were measured (3 mice per group; 1 of 2 experiments). In separate experiments, (**b**) respiratory rates, minute volumes, and enhanced pause (PenH) were measured on the indicated days (5 mice per group per day; 1 of 2 experiments). All error bars represent the standard deviation, and * *P* < 0.05, *** *P* < 0.001, **** *P* < 0.0001.

The inflammatory factors seen in the lung following systemic IL-2 administration could originate from other sites. However, when compared to the response seen with i.p. administration, an even more robust response was seen in the lung when IL-2C was administered intranasally (**Fig 5A**). This supports the conclusion that beyond the known ability of IL-2 to stimulate vascular leak and pulmonary edema [46, 48], the lung environment is extremely sensitive to rapid IL-2-dependent induction of inflammatory cytokines and chemokines, even in the absence of infection. As the 2 μg dose of IL-2C may deliver sustained IL-2 signaling not typically achieved during immune responses, we titrated the amount of IL-2 used. The pro-inflammatory impact of the IL-2C was proportional to the dose administered, with significant pro-inflammatory effects in analytes such as CCL2 arising with even 0.5 μg of IL-2 and broad effects seen at 1 μg. The impact of the IL-2C was abrogated by pre-treating hosts with blocking antibody against CD122 (**S3B Fig**), confirming that IL-2 itself rather than potential contaminants in reagents was responsible for the inflammatory impact.

In agreement with previous observations [45], IL-2 and IL-2C treatment also caused the expansion of several major leukocyte populations in the spleen (**S3C Fig**). Some of these patterns were also seen in the lung. In particular, IL-2C treatment dramatically expanded NK cells and to a lesser extent CD8 and CD4 T cells as well as inflammatory CD45+ MHC-II+ CD11b+ Ly6C+ APC [49] (**S3C and S3D Fig**). Given the dramatic effect of IL-2C administration on the lung environment, we assessed pulmonary mechanics in uninfected animals receiving IL-2C. When compared to untreated controls, the IL-2C-driven inflammatory response correlated with decreased respiratory function (**Fig 5B**).

**Fig 6:**
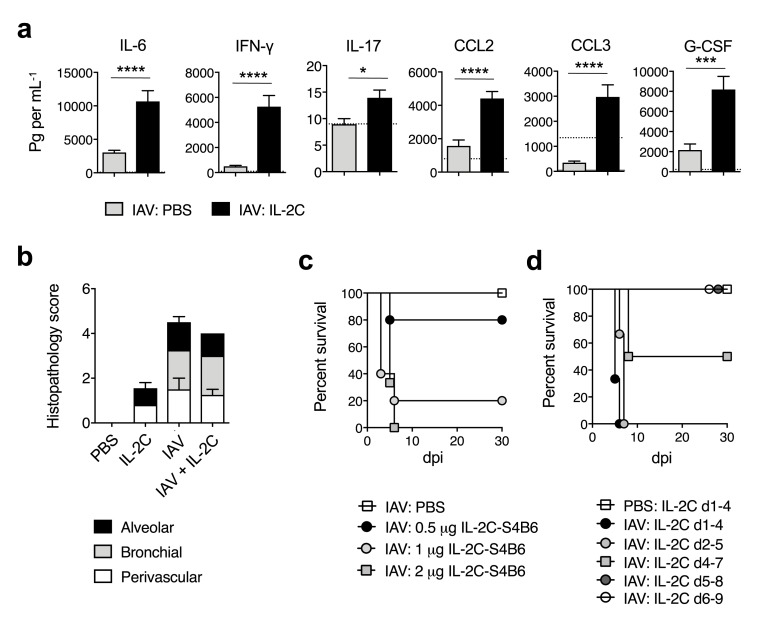
Early IL-2 synergizes with IAV to drive lethal inflammatory responses. Uninfected and sublethal 0.2 LD_50_ A/PR8-OVA_II_ infected BALB/c mice were treated with PBS or IL-2Cs containing 2 μg of IL-2 for 3 days. On day 4, inflammatory responses in lung homogenates were measured. The levels of cytokines and chemokines detected are shown in (**a**) (3 mice per group; 1 of 3 experiments). The dashed line in bar graphs represents the level of analyte detected with IL-2C treatment alone. In separate experiments, lungs were evaluated blindly and scored for inflammation in the bronchi, blood vessels, and alveoli (**b**). IL-2C treatment with the indicated amount of IL-2 was extended to 4 days (**c**) or given on the indicated days (**d**) and mortality monitored (3–5 mice per group; 1 of 3 experiments). All error bars represent the standard deviation, and * *P* < 0.05, *** *P* < 0.001, **** *P* < 0.0001.

We next tested whether and how IL-2 impacts the outcome of primary IAV infection by treating unprimed WT mice with IL-2C for the first 3 days of a 0.2 LD_50_ A/PR8 challenge. Infected mice also treated with IL-2C displayed higher levels of a broad array of cytokines and chemokines in the lung compared to mice only infected with IAV or mice only treated with IL-2C **(Fig 6A),** indicating strong synergy between infection-induced and IL-2-dependent inflammatory pathways. However, histopathological changes were not appreciably enhanced in mice receiving IAV and IL-2C versus mice treated with only IAV or only with IL-2C (**Fig 6B** and **S4 Fig**), agreeing with the lack of histological changes in recipients of WT or *Il2*^*-/-*^ memory CD4 T cells following IAV infection (Fig 3). Nevertheless, IL-2C treatment for 4 instead of 3 days resulted in acute death of infected mice, even when lower amounts of IL-2 were used (**Fig 6C**). In marked contrast, when IL-2C treatment was initiated at later time-points (5 to 9 dpi) that coincide with the onset of viral clearance, all mice survived (**Fig 6D**). Thus, IL-2 signals delivered early but not at later timepoints of infection when T cell effectors reach their peak, potently enhance acute IAV-induced recruitment and/or activation of inflammatory cells in the lung and transform a mild illness to a fatal infection.

### IL-2 induced NK cells are major contributors of lung inflammation and fatal outcomes

**Fig 7:**
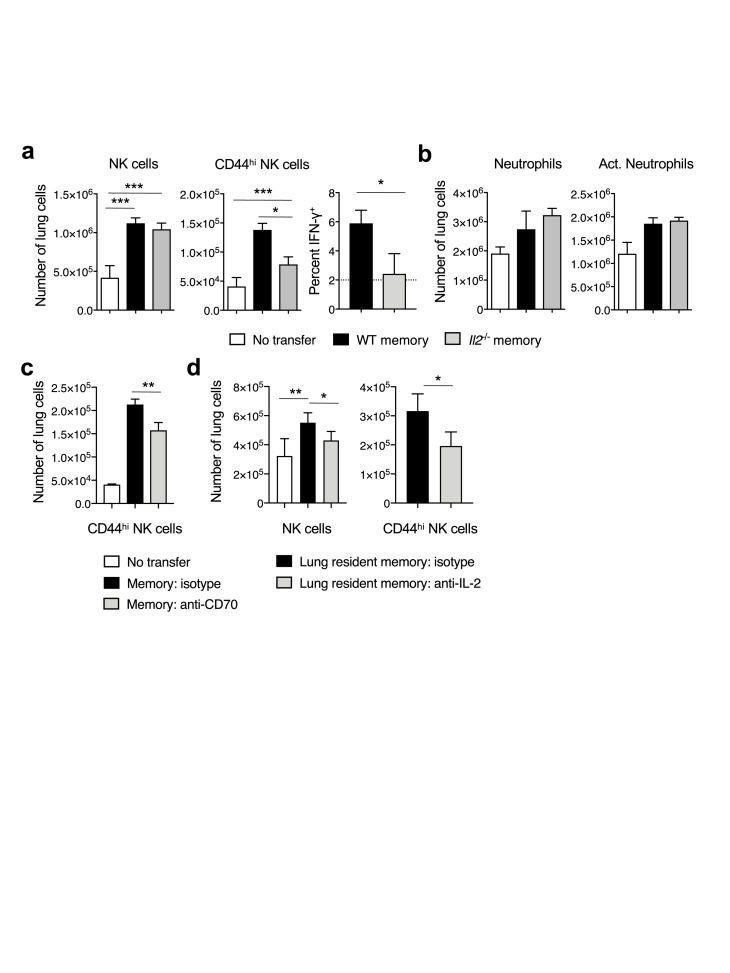
Memory CD4 T cell derived IL-2 induces NK cell activation. Unprimed BALB/c hosts received WT or *Il2*^*-/-*^ memory DO11.10 CD4 T cells and were infected with 2 LD_50_ dose of A/PR8-OVA_II_ virus. On day 4, (**a**) total numbers (left), activated (center), and IFN-γ cytokine producing NK cells (right) and (**b**) total and activated neutrophils in the lung were assessed by flow cytometry (3–5 mice per group; 1 of 3 experiments). In (**c**), the number of activated NK cells in the lung of WT memory CD4 T cell recipients in the presence and absence of CD70 blocking antibody (Ab) was determined 4 dpi (4 mice per group; 1 of 2 experiments). In separate experiments, lung resident memory CD4 T cells were isolated on 21 dpi from IAV-primed mice and 1 x 10^6^ administered through the intranasal route. Recipient mice were infected with 2 LD_50_ of A/PR8 and on day 4, (**d**) total numbers (left) and activated (right) NK cells in the lung were assessed (4 mice per group; 1 of 2 experiments). All error bars represent the standard deviation, and * *P* < 0.05, ** *P* < 0.01, *** *P* < 0.001.

Since NK cells and neutrophils were enhanced by IL-2C, and are prominently involved in driving IL-2-dependent vascular leak [50, 51], we determined their involvement in the IL-2-induced inflammatory response. We first characterized the dynamics of NK and neutrophil responses in the lung at 3 dpi in naïve recipients of WT or *Il2*^*-/-*^ memory CD4 T cells, or in controls not receiving cell transfer. While total numbers of NK cells in both WT or *Il2*^*-/-*^ memory CD4 T cell recipients were similar, significantly more activated (CD44^hi^ and IFN-γ^+^) NK cells were detected in WT memory CD4 T cell recipients (**Fig 7A**). No differences in total neutrophil number or activation (SSC^hi^ CD69^hi^) were seen (**Fig 7B**). Significantly more activated NK cells were also detected at 4 dpi in isotype antibody-treated recipients of WT memory CD4 T cells than in recipients treated with CD70 blocking antibody (**Fig 7C**), in which reduced levels of paracrine IL-2 are detected (Fig 4B).

We next evaluated the ability of lung-resident memory CD4 T cells isolated from IAV-primed mice to modulate NK cell responses in the presence and absence of IL-2 neutralizing antibody to ensure that the observations made with *in vitro*-primed memory populations accurately recapitulate elements of the IAV-primed responses. Polyclonal IAV-primed lung-resident memory CD4 T cells were transferred intranasally to naïve recipients as previously described [12] and lung NK cell responses following IAV infection analyzed on 4 dpi. Both the number of total NK cells as well and activated NK cells were reduced when IL-2 was neutralized in lung-resident memory CD4 T cell recipients (**Fig 7D**), mirroring findings obtained with *in vitro*-primed memory CD4 T cells.

Finally, we tested whether memory CD4 T cell-derived IL-2 could impact NK cell responses in lungs imprinted by prior IAV infection [52] rather than in the models above assessing memory CD4 T cell responses in otherwise naive mice. We transferred WT memory CD4 T cells to naive hosts and primed with IAV. At 60 dpi, cognate peptide was administered intranasally to recall the donor CD4 T cells in the lung [31]. We analyzed lung NK cells 4 days after peptide administration in mice treated with isotype or IL-2 neutralizing antibodies and found a reduction in activated, but not total NK cells in the absence of IL-2 signaling (**S5 Fig**). Thus, using multiple approaches, we find that IL-2 production from memory CD4 T cells following TcR stimulation significantly impacts the acute activation profile of NK cells in the lung.

**Fig 8:**
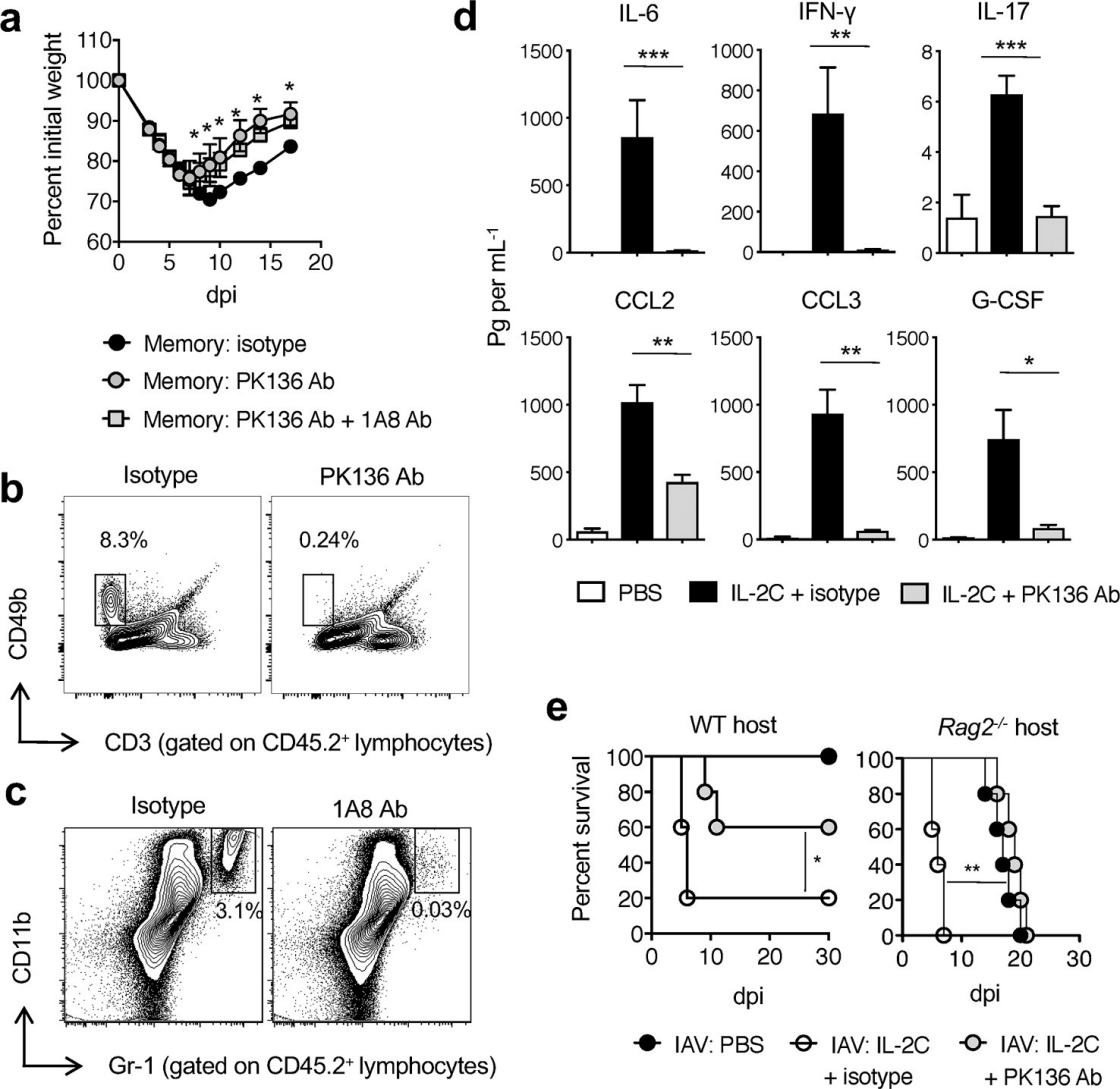
NK cells are major contributors to IL-2-induced morbidity and mortality. OT-II CD4 memory T cell driven morbidity (**a**) following 2 LD_50_ A/PR8-OVA_II_ infection was assessed in C57BL/6 mice in the absence and presence of NK cell (PK136) depleting antibody only or in addition to neutrophil (1A8) depleting antibody (3 mice per group; 1 of 3 experiments). Representative staining confirming the depletion of NK cells (**b**) and neutrophils (**c**) is shown. In (**d**), IL-2Cs containing 2 μg of IL-2 were administered to naive C57BL/6 mice for 3 days in the presence of isotype or NK cell (PK136) depleting antibody. Inflammatory responses in lung homogenates were measured on day 4 (n = 3 mice per group in replicate experiments). In (**e**), A/PR8-OVA_II_ infected (2500 EID_50_) WT C57BL/6 and *Rag2*^*-/-*^ mice were treated with PBS or IL-2Cs containing 2 μg of IL-2 for 3 days together with isotype or NK depleting antibody and survival monitored (3 mice per group; 1 of 3 experiments). All error bars represent the standard deviation, and * *P* < 0.05, ** *P* < 0.01, *** *P* < 0.001, **** *P* < 0.0001.

Given the results above and observations that NK cells can maximize neutrophil responses *in vivo* [53], we next depleted NK cells in naïve mice receiving WT memory CD4 T cell prior to IAV challenge to determine whether and how NK cells impact the outcome of infection. NK cell depletion resulted in reduced weight loss and earlier recovery (**Fig 8A** and **8B**), phenocopying the improved outcomes seen in *Il2*^*-/-*^ versus WT memory CD4 T recipients depicted in Fig 3, as well that of animals treated with CD70 blocking antibody in Fig 4. Even though they are known to contribute to IL-2 driven vascular leak, additional depletion of neutrophils did not appreciably alter the course of IAV infection (**Fig 8A** and **8C**).

The involvement of NK cells in hampering CD4 T cell memory mediated protection prompted us to determine the extent to which IL-2-induced NK cell responses directly contribute to the production of acute inflammatory factors. To directly test this, we depleted NK cells prior to treating naïve mice with IL-2C and measured inflammatory cytokines and chemokines in the lungs. NK cell depletion decreased levels of all cytokines and chemokines enhanced by IL-2C treatment during IAV infection (**Fig 8D**), demonstrating the IL-2-dependent ability of NK cells to markedly shape acute pulmonary inflammation. Finally, to firmly establish a detrimental role for IL-2-driven NK cell responses in the lung during IAV infection, we depleted NK cells in naïve mice prior to IL-2C administration and sublethal IAV challenge. Remarkably, NK cell depletion protected IAV-challenged WT and *Rag2*^-/-^ mice from acute IL-2C-dependent death (**Fig 8E**).

In summary, our results using both reductionist models and analysis of intact mice responding to IAV strongly suggest that IL-2 produced by memory CD4 T cells can drive enhanced inflammatory responses in the lung. A major mechanism involved in driving this response is the IL-2-dependent promotion of early NK cell activity that can detrimentally impact the resolution of IAV challenge.

## Discussion

Defining the most incisive correlates of protective memory T cells in a disease-specific manner is critical to improve monitoring of clinical responses and to promote tailored attributes of T cells induced by vaccination. Fully understanding the impact of tissue environments on the outcome of memory T cell recall is equally important but is poorly understood. Here, we show that during memory CD4 T cell-mediated protective immune responses against IAV, their IL-2 production induces enhanced proinflammatory cytokine production and NK cell responses leading to delayed recovery and compromised lung function. Welsh and collaborators have noted the rheostat nature of NK cell function after viral infection and the pathogenic impact of too many NK cells during medium and high dose LCMV infection [54]. In the setting of IAV, the beneficial versus detrimental impact of NK cells and neutrophil responses remains controversial [55, 56]. Our results suggest that the relative amount of IL-2 available during the early phases of the immune response can be an important contextual determinant in regulating the positive versus negative impact of these innate subsets.

Our findings suggest that, at least in the case of IAV infection, the ability of memory CD4 T cells to produce high levels of IL-2 in conjunction with IFN-γ cannot alone be taken as an indicator of superior protective potential. They do not, however, necessarily contradict the general concept that multi-cytokine-producing Th1 memory cells are more protective than cells only able to secrete IFN-γ [57]. Indeed, polyfunctional memory CD4 T cells express a unique molecular signature compared to single IFN-γ producers [58]. Moreover, there are very few differences in the transcriptome of wild-type and IL-2-deficient memory CD4 T cells responding against IAV [15]. These observations support the concept that key protective molecular signatures of multi-functional CD4 memory may be independent of IL-2 expression during antigenic recall, and as we have shown, IL-2 may instead have its positive activity primarily at the effector stage to promote CD4 T cell memory generation [19].

Although we observed similar impacts using TcR transgenic and polyclonal CD4 T cells, a comprehensive understanding of how and when IL-2 produced by CD4 T cell populations impacts infection requires further study. For example, CD4 T cells with different TcR specificities have been shown to produce different amounts of IL-2 upon restimulation with cognate IAV peptides [59], and both higher doses of antigen and higher avidity T cell responses lead to greater IL-2 production [60]. These findings indicate that responses against certain antigens may be more or less impacted by the mechanisms described here. Furthermore, optimal CD4 T cell-mediated IAV clearance requires synergy between many different specialized subsets [61–63]. Whether IL-2 from subsets other than Th1-like cells similarly impacts recall against IAV requires exploration, but we stress that the majority of CD4 T cells responding to IAV fit general Th1-like criteria.

Additional studies are also required to determine if IL-2 production from memory CD4 T cells similarly impacts responses against other pathogens, as well as in other tissues. Adjuvant effects following IL-2 administration observed during systemic viral infection support that memory CD4 T cell-derived IL-2 may have similar proinflammatory effects in this setting [64]. However, that we found a stronger impact of IL-2 on inflammation in the lung than in the serum, even when IL-2 was administered systemically, suggests the lung and responses against respiratory infections may be particularly sensitive to IL-2. Indeed earlier studies found exogenous IL-2 administration to cause cellular proliferation only in specific tissues, including the lung [65]. We speculate that IL-2 production from vaccine-induced memory CD4 T cells may in some cases be a component of their immunopathological impact, such as that observed during chronic LCMV infection [22].

We previously found that blocking CD70 during IAV priming dramatically decreased the number of memory CD4 T cells formed [19]. The impact on memory generation was due to a reduction of CD27-dependent autocrine IL-2 production induced by cognate interactions with CD70^+^ dendritic cells. Here, we found CD70 blockade to reduce inflammation driven by memory CD4 T cells responding to IAV. Interestingly, CD70 blockade significantly decreased IL-2 production as well as IFN-γ production by memory CD4 T cells. While higher levels of IFN-γ have been implicated in increasing susceptibility to primary IAV infection [66], we have shown that IFN-γ signaling does not significantly impact the ability of memory CD4 T cells to drive protective inflammatory responses against IAV [26, 31]. Thus, while we cannot formally rule out that changes in IFN-γ and other factors have no impact on the improved responses seen with blocking CD70, our data strongly supports the hypothesis that changes in IL-2 production play a central role in this process. Collectively, our observations suggest that the CD27-CD70 pathway may be targeted as a temporal rheostat to modulate CD4 T cell immunity: during priming, targeting the CD70-CD27 pathway to enhance IL-2 production can serve to boost the efficiency of memory generation, while early during recall, blocking this pathway may help to restrain IL-2-driven inflammation originating from memory CD4 T cells.

Given the potent and broad-ranging proinflammatory activity of IL-2, we predict that mechanisms must be in place to limit its impact. The lung may be particularly sensitive to IL-2-driven inflammation because of endothelial cell expression of functional IL-2 receptors [46]. We found previously that while CD4 T cell effectors responding to IAV in the spleen and draining lymph nodes produce high levels of IL-2, only about 10% of cells responding in the lung at the peak of the anti-viral response demonstrate robust IL-2 production [10]. This is in stark contrast to the strong IL-2 production potential observed from CD4 T cells that develops in the lungs only after the clearance of virus as well as at memory timepoints [12, 67, 68]. We propose that these findings reflect two distinct kinds of control on IL-2 production by CD4 T cells. First, control of T cell cytokine production appears to be an inherent property of the lung environment [69], which may serve to buffer against IL-2-induced inflammation to maintain maximal pulmonary function. Second, further controls on IL-2 production are likely induced by infection. For example, PDL-1, which dramatically impedes IL-2 production by activated T cells upon ligation of PD-1 [70, 71], is strongly upregulated in the lung early in the course of IAV infection [72, 73]. The extent to which the capacity of lung IAV-specific CD4 T cells to produce high levels of IL-2 during the transition to memory following viral clearance [67] is resultant from downmodulation of inhibitory molecules such as PDL-1 versus CD4 T cell-intrinsic mechanisms warrants further study. Interestingly, IL-2 production by CD4 T cells is needed to promote production of the anti-inflammatory cytokine IL-10 by CD8 T cells responding to IAV [74], which make little of their own IL-2 during heterosubtypic responses [8]. IL-10^+^ CD8 T cells protect mice against lethal IAV-induced inflammation in some [75] but not other [32] models. Induction of IL-10^+^ CD8 T cells by CD4 T cell-derived IL-2 may thus act as further layer of buffering against damaging inflammation during respiratory infections. The impact of these restraining mechanisms likely lessens following pathogen clearance.

Therapeutic delivery of IL-2, such as by systemic IL-2C treatment using the S4B6 clone and 1–2 μg of recombinant IL-2 has seen increasing use in experimental models to modulate CD8 T cell and NK cell populations *in vivo* to improve responses against pathogens and cancers [43]. Our results stress the importance of careful evaluation of how these treatment regimens impact inflammatory environments when interpreting experimental outcomes. Furthermore, we stress that while IL-2 production can be boosted or restored by the systemic blockade of checkpoint inhibitors [76, 77], how production of IL-2 by T cells responding to respiratory infection impacts patient outcome in such settings, including cancer therapy, remains to be determined. Our results argue the clinical use of IL-2 and engineered IL-2 and IL-15 [78] should be viewed with caution, given the fatal outcomes observed when IL-2C treatment synergized with mild, low-dose, IAV infection.

## Methods

### Ethics statement

Experimental animal procedures were conducted in accordance with guidelines outlined by the Office of Laboratory Animal Welfare (OLAW), National Institute of Health, USA. Protocols were approved by the Animal Care and Use Committee at Trudeau Institute (Saranac Lake, NY) protocol 00–33, the Institutional Animal Care and Use Committee of the University of Massachusetts Medical School (Worcester, MA) protocol A-2198, and the University of Central Florida (Orlando, FL) protocol 18–30.

### Mice

Naïve CD4^+^ T cells were obtained from 5 to 8-week-old male or female DO11.10 Thy1.2/Thy1.1 and *Il2*^*-/-*^ DO11.10 Thy1.2/Thy1.1 mice originally provided by A. Abbas (UCSF). Recipients of cell transfers were male BALB/c.Thy1.2 or BALB/c.Thy1.1, nude, J_H_D, or SCID mice that were at least 8 weeks old. In some experiments, naïve CD4 T cells were obtained from 5 to 8-week-old male OT-II Thy1.2/1.1 mice and C57BL/6 and *Rag2*^-/-^ male recipients were used. Nude, *Rag2*^-/-^, J_H_D, and SCID mice were purchased Charles River, Taconic, or Jackson Laboratories. All other mice were obtained from Jackson Laboratories or the breeding facility at Trudeau Institute, the University of Massachusetts Medical School, or the University of Central Florida.

### CD4 T cell isolation and *in vitro*-primed memory generation

Naïve CD4^+^ T cells were obtained from pooled spleen and peripheral lymph nodes as previously described [24]. Briefly, cells were purified by nylon wool and percoll density gradient separation. CD4 T cells were isolated by positive CD4 MACS selection (Miltenyi). Resulting CD4^+^ cells routinely expressed a characteristic naive phenotype (small size, CD62L^hi^, CD44^lo^ and CD25^lo^) >97% TcR^+^. T_H_1-polarized effectors were generated *in vitro* as described [5]. Briefly, naïve WT or *Il2*^*-/-*^ CD4 T cells were cultured with an equal number of irradiated APC (2x10^5^ per mL) in the presence of exogenous IL-2 (20 ng per mL), 2 ng per mL IL-12 (Peprotech), 10 μg per mL anti-IL-4 antibody (11B11; Bioxcell), and 5 μM OVA_II_ peptide. *In vitro*-primed memory cells were obtained by thoroughly washing effector cultures at 4 days and re-culturing the cells in fresh media for at least 3 days in the absence of Ag and exogenous cytokines. Live cells were isolated by Lympholyte separation (Cedarlane). All donor CD4 T cells were adoptively transferred in 200 μl phosphate buffered saline (PBS) by intravenous (i.v.) injection. A number of donor cells previously determined to protect against lethal IAV infection, 5 x 10^6^, was transferred. In some experiments, donor CD4 T cells were labeled with CFSE, as previously described [10], prior to adoptive transfer to monitor *in vivo* proliferation. In some experiments, lung resident memory CD4 T cells were isolated from IAV-primed mice and 1 x 10^6^ adoptively transferred to recipient mice through the intranasal route as previously described [12].

### Virus stocks and infections

Influenza A/Puerto Rico/8/1934 (PR8) (H1N1) originating from stocks prepared at the Trudeau Institute and in use in experiments since 1997, A/PR8-OVA_II_ (H1N1) from stock obtained from P. Doherty at St Jude’s Children’s Hospital [79], and the cold-adapted attenuated strain A/Alaska/6/1977 CR-29, (H3N2) virus kindly provided by S. Epstein, NIH were produced in the allantoic cavity of embryonated hen eggs at the Trudeau Institute and the lethal dose (LD_50_), egg infective dose (EID_50_) or tissue culture infective dose (TCID_50_) characterized. Mice were infected intranasally under light isoflurane anesthesia (Webster Veterinary Supply) with the indicated doses of virus in 50 μl PBS and morbidity and mortality monitored. Donor cell injection and viral infection occurred on the same day. In some experiments, 5 μg of cognate peptide was administered intranasally to mice that had received donor memory CD4 T cells and IAV primed 60 days prior. The recovery day, or the day when animals began to regain weight following infection, was also determined.

### Detection of IAV titer

Pulmonary viral titer was determined by quantitation of viral RNA. RNA was prepared from whole lung homogenates using TRIzol (Sigma-Aldrich), and 2.5 μg of RNA was reverse transcribed into cDNA using random hexamer primers and Superscript II Reverse Transcriptase (Invitrogen). Quantitative PCR was performed to amplify the acidic polymerase (PA) gene of A/PR8-OVA_II_ using an ABI Prism 7700 Sequence Detector (Applied Biosystems) with 50 ng of cDNA per reaction and the following primers and probe: forward primer, 5'-CGGTCCAAATTCCTGCTGA-3'; reverse primer, 5'CATTGGGTTCCTTCCATCCA-3'; probe, 5'-6-FAM-CCAAGTCATGAAGGAGAGGGAATACCGCT-3'. Data were analyzed with Sequence Detector v1.7a (Applied Biosystems). The copy number of the PA gene per 50 ng of cDNA was calculated using a PA-containing plasmid of known concentration as a standard. The number of copies of PA gene per lung is presented.

### Cytokine complex, receptor blockade, and depleting antibody treatments

Mice were treated for the indicated days with injections of cytokine or cytokine: anti-cytokine monoclonal antibody complexes. For IL-2 complexes (IL-2C), mice received 2 μg per day of recombinant IL-2 (eBioscience) premixed with 20 μg of anti-mouse IL-2 monoclonal antibody clone S4B6-1 (S4B6) (BD Pharmingen). In certain experiments, the amount of IL-2 in the complexes was varied, as indicated. Complexes were incubated at room temperature for 20 minutes (min.) before intraperitoneal (i.p.) injection in 200 μL of PBS. IL-2C in 50 μL of PBS were also administered intranasally (i.n.). When IL-2 was administered as free cytokine, animals were treated with 20 μg per day in 200 μL of PBS injected i.p.

For some experiments, mice were treated as indicated with 0.25 mg per day of anti-CD122 (IL-2 Rβ) antibody (5H4) to block IL-2 signaling, 0.25 mg per day anti-IL-2 antibodies (S4B6 and JES6-1A12) to neutralize IL-2, 0.5 mg per day of anti-CD70 antibody (FR-70) to block CD70 signaling, 0.25 mg per day of anti-NK1.1 (PK136) to deplete NK cells, 0.5 mg of anti Ly-6G Ab to deplete neutrophils (1A8), or with appropriate isotype control antibody (all Bioxcell). Antibody was delivered by i.p. injection in 200 μL of PBS.

### Tissue preparation and flow cytometry

At different time points after virus infection, blood and lungs were obtained from euthanized animals for Luminex multiplex analysis. Lungs were harvested and homogenized in RPMI 1640 media supplemented with 2mM L-glutamine, 100 IU penicillin, 100 μg/mL streptomycin (Invitrogen), 10 mM HEPES (Research Organics), 50 μM 2-mercaptoethanol (Sigma-Aldrich) and 7.5% fetal bovine serum (Hyclone) and serum collected from blood.

Alternatively, for flow cytometry, mice were euthanized by cervical dislocation followed by exsanguinated by perforation of the abdominal aorta. Lungs were perfused by injecting 10 ml of PBS in the left ventricle of the heart. Lungs and spleen were prepared into single cell suspensions by mechanical disruption of organs and passage through a nylon membrane. Flow cytometry was performed as previously described [24] using fluorochrome-labeled antibodies at manufacturer’s recommended dilutions for surface staining including anti-Thy1.1 (OX-7), anti-Thy1.2 (53–2.1), anti-CD4 (RM4.5 and GK1.5), anti-CD8 (53–6.7), anti-CD45.2 (104), anti-γδ TcR (GL3), anti-β TCR (H57-597), anti-CD3 (17A2), anti-CD49d (R1-2), anti-CD25 (PC61), anti-CD44 (1M7.8.1), anti-CD69 (H1.2F3), anti-CD11b (M1/70), anti-Gr-1 (RB6-8C5), anti-MHC-II (M5/114.15.2), and anti-Ly6C (HK1.4). In some experiments, tissue resident memory CD4 T cells were identified by intravenous administration of 3 μg of fluorochrome-labeled antibody 3 minutes prior to euthanasia and tissue harvest [12].

Intracellular cytokine staining was performed as previously described [19]. Briefly, cells were treated with PMA and Ionomycin for 4 hours or stimulated overnight with cognate peptide presented by APC, with Brefeldin A added after 2 h. Cells were then surface stained, fixed for 20 min. in 4% paraformaldehyde, and permeabilized by 10 min. incubation in 0.1% saponin before staining for cytokine by the addition of anti-IFN-γ and anti-IL-2 fluorescently labeled antibodies. Analysis was performed using FACS Canto II and LSRII instruments (BD Biosciences) and FlowJo (Tree Star) analysis software.

### Detection of inflammatory cytokines and chemokines and BAL albumin

Levels of cytokines and chemokines in lung homogenates or serum were determined using mouse multiplex kits (Invitrogen and Millipore) read on a Bio-Plex Multiplex 200 Luminex reader (Bio-Rad). Levels of serum albumin in the BAL fluid were determined using a Mouse Albumin ELISA Quantification Kit as per manufacturer’s instructions (Bethyl Laboratories Inc.).

### Histology

For assessment of immunopathology following viral infection and IL-2C treatment, lungs lobes were isolated and immediately fixed in 10% neutral buffered formalin. Lung samples were subsequently processed, embedded in paraffin, sectioned, placed on L-lysine-coated slides, and stained with Hematoxylin and Eosin (H&E) using standard histological techniques. Sections were graded blindly from 0 to 4, for the extent of inflammatory cell infiltration and damage of bronchi, arteries or alveoli by a certified pathologist (S. Sell).

### Measurement of pulmonary mechanics

Non-invasive whole-body plethysmography (WBP) (Buxco) was employed to measure respiratory rates (breaths/min.), minute volumes (mL/min.), and enhanced pause PenH, on conscious, unrestrained animals following IAV infection and IL-2C treatment. The minute volume is defined as the volume of air exchanged during a 1-min. interval and is calculated as follows [respiratory rate X tidal volume].

### Statistical analysis

Group sizes of n = 3 to 15 were employed. Unpaired, two-tailed, Students *t*-tests, ∞ = 0.05, were used to assess whether the means of two normally distributed groups differed significantly. One-way ANOVA analysis with Bonferroni’s multiple comparison post-test was employed to compare multiple means. Two-way ANOVA analysis with repeated measures was also employed in some experiments. The Log Rank test was used to test for significant differences in Kaplan-Meier survival curves. All error bars represent the standard deviation. Significance is indicated as * *P* < 0.05, ** *P* < 0.005, *** *P* < 0.001, **** *P* < 0.0001.

## Supporting information

S1 Fig Engraftment and response of WT and *Il2*^*-/-*^ memory CD4 cells in congenic adoptive transfer recipients. Unprimed BALB/c recipients of WT or *Il2*^*-/-*^ memory DO11.10 CD4 T cells were challenged with a 2 LD_50_ dose of A/PR8-OVA_II_ virus. On indicated days, engraftment and expansion of donor cells was assessed in spleens, draining lymph nodes (dLN), and lungs of recipient mice. Representative donor cell expression of the congenic marker and CD4 on gated lymphocytes is shown in (**a**) and donor cell expansion via loss of CFSE shown in (**b**). Percentages represent the frequency of donors among total lymphocytes (3–5 mice per group; 1 of 3 experiments).
(EPS)

S2 Fig IL-2 production is not needed for IAV-specific memory CD4 T cell-mediated protection in T and B cell deficient hosts. The indicated unprimed immunodeficient recipient hosts received WT or *Il2*^*-/-*^ memory DO11.10 CD4 T cells and were challenged with a 2500 EID_50_ dose of A/PR8-OVA_II_ virus. Morbidity was monitored (3–5 mice per group; 1 of 3 experiments). All error bars represent the standard deviation and * *P* < 0.05.
(EPS)

S3 Fig IL-2 drives potent inflammatory responses in the lung. Naive BALB/c mice were treated with PBS, 20 μg of IL-2, or IL-2Cs containing 2 μg of IL-2 for 3 days. On day 4, inflammatory responses in lung homogenates and serum were measured (**a**) (summary from 3 experiments containing 3 mice per group). In separate experiments, mice were treated with IL-2C containing the indicated amount of IL-2 without or with anti-CD122 antibody to block the IL-2R. Inflammatory responses in lung homogenates were measured on d4 (**b**) (3 mice per group; 1 of 2 experiments). Lymphocyte populations in the spleen and lung of IL-2 or IL-2C treated animals were enumerated and compared to control mice (**c**). The frequency of inflammatory CD45^+^ MHC-II^+^ CD11b^+^ Ly6C^+^APC were also determined in the lung of mice treated with IL-2C or PBS alone (**d**). All error bars represent the standard deviation, and * *P* < 0.05, ** *P* < 0.01, *** *P* < 0.001, **** *P* < 0.0001.
(EPS)

S4 Fig IL-2 contributes to alveolar and perivascular histopathology. Uninfected and low-dose, sublethal 0.2 LD_50_ A/PR8-OVA_II_ infected BALB/c mice were treated with PBS or IL-2Cs containing 2 μg of IL-2 for 3 days. Representative photomicrographs of H & E stained tissue sections of lungs on 4 dpi are shown, Br: bronchus; Ar: artery.
(EPS)

S5 Fig Memory CD4 T cell derived IL-2 induces NK cell activation in IAV primed environments. Unprimed BALB/c hosts received WT memory CD4 T cells and were infected with 0.5 LD_50_ A/PR8-OVA_II_ virus. On day 60 and 62 post priming, 5 μg of cognate peptide was administered and total numbers and activated NK cells assessed by flow cytometry (4 mice per group) and * *P* < 0.05.
(EPS)

## References

[1] Malek TR. The biology of interleukin-2. Annu Rev Immunol. 2008;26:453–79. 10.1146/annurev.immunol.26.021607.090357 18062768

[2] Marcais A, Viel S, Grau M, Henry T, Marvel J, Walzer T. Regulation of mouse NK cell development and function by cytokines. Front Immunol. 2013;4:450 10.3389/fimmu.2013.00450 24376448 PMC3859915

[3] Laidlaw BJ, Craft JE, Kaech SM. The multifaceted role of CD4(+) T cells in CD8(+) T cell memory. Nat Rev Immunol. 2016;16(2):102–11. 10.1038/nri.2015.10 26781939 PMC4860014

[4] Sojka DK, Bruniquel D, Schwartz RH, Singh NJ. IL-2 secretion by CD4+ T cells in vivo is rapid, transient, and influenced by TCR-specific competition. J Immunol. 2004;172(10):6136–43. 10.4049/jimmunol.172.10.6136 15128800

[5] McKinstry KK, Golech S, Lee WH, Huston G, Weng NP, Swain SL. Rapid default transition of CD4 T cell effectors to functional memory cells. J Exp Med. 2007;204(9):2199–211. 10.1084/jem.20070041 17724126 PMC2118696

[6] Dienz O, Eaton SM, Krahl TJ, Diehl S, Charland C, Dodge J, et al Accumulation of NFAT mediates IL-2 expression in memory, but not naive, CD4+ T cells. Proc Natl Acad Sci U S A. 2007;104(17):7175–80. 10.1073/pnas.0610442104 17438271 PMC1855411

[7] Talker SC, Stadler M, Koinig HC, Mair KH, Rodriguez-Gomez IM, Graage R, et al Influenza A Virus Infection in Pigs Attracts Multifunctional and Cross-Reactive T Cells to the Lung. J Virol. 2016;90(20):9364–82. 10.1128/JVI.01211-16 27512056 PMC5044846

[8] Strutt TM, McKinstry KK, Kuang Y, Finn CM, Hwang JH, Dhume K, et al Direct IL-6 Signals Maximize Protective Secondary CD4 T Cell Responses against Influenza. J Immunol. 2016;197(8):3260–70. 10.4049/jimmunol.1600033 27647834 PMC5101150

[9] Seder RA, Darrah PA, Roederer M. T-cell quality in memory and protection: implications for vaccine design. Nat Rev Immunol. 2008;8(4):247–58. 10.1038/nri2274 18323851

[10] Strutt TM, McKinstry KK, Kuang Y, Bradley LM, Swain SL. Memory CD4+ T-cell-mediated protection depends on secondary effectors that are distinct from and superior to primary effectors. Proc Natl Acad Sci U S A. 2012;109(38):E2551–60. 10.1073/pnas.1205894109 22927425 PMC3458385

[11] Teijaro JR, Turner D, Pham Q, Wherry EJ, Lefrancois L, Farber DL. Cutting edge: Tissue-retentive lung memory CD4 T cells mediate optimal protection to respiratory virus infection. J Immunol. 2011;187(11):5510–4. 10.4049/jimmunol.1102243 22058417 PMC3221837

[12] Strutt TM, Dhume K, Finn CM, Hwang JH, Castonguay C, Swain SL, et al IL-15 supports the generation of protective lung-resident memory CD4 T cells. Mucosal Immunol. 2018;11(3):668–80. 10.1038/mi.2017.101 29186108 PMC5975122

[13] Purwar R, Campbell J, Murphy G, Richards WG, Clark RA, Kupper TS. Resident memory T cells (T(RM)) are abundant in human lung: diversity, function, and antigen specificity. PLoS ONE. 2011;6(1):e16245 10.1371/journal.pone.0016245 21298112 PMC3027667

[14] Oja AE, Piet B, Helbig C, Stark R, van der Zwan D, Blaauwgeers H, et al Trigger-happy resident memory CD4(+) T cells inhabit the human lungs. Mucosal Immunol. 2018;11(3):654–67. 10.1038/mi.2017.94 29139478

[15] Kumar BV, Ma W, Miron M, Granot T, Guyer RS, Carpenter DJ, et al Human Tissue-Resident Memory T Cells Are Defined by Core Transcriptional and Functional Signatures in Lymphoid and Mucosal Sites. Cell Rep. 2017;20(12):2921–34. 10.1016/j.celrep.2017.08.078 28930685 PMC5646692

[16] Wilkinson TM, Li CK, Chui CS, Huang AK, Perkins M, Liebner JC, et al Preexisting influenza-specific CD4+ T cells correlate with disease protection against influenza challenge in humans. Nat Med. 2012;18(2):274–80. 10.1038/nm.2612 22286307

[17] Lin Y, Zhao YB, Zeng XJ, Lu CP, Liu YJ. Complete genome sequence of an H3N2 canine influenza virus from dogs in Jiangsu, China. J Virol. 2012;86(20):11402 10.1128/JVI.01946-12 22997421 PMC3457169

[18] Dooms H, Wolslegel K, Lin P, Abbas AK. Interleukin-2 enhances CD4+ T cell memory by promoting the generation of IL-7R alpha-expressing cells. J Exp Med. 2007;204(3):547–57. 10.1084/jem.20062381 17312008 PMC2137906

[19] McKinstry KK, Strutt TM, Bautista B, Zhang W, Kuang Y, Cooper AM, et al Effector CD4 T-cell transition to memory requires late cognate interactions that induce autocrine IL-2. Nat Commun. 2014;5:5377 10.1038/ncomms6377 25369785 PMC4223689

[20] Brincks EL, Roberts AD, Cookenham T, Sell S, Kohlmeier JE, Blackman MA, et al Antigen-specific memory regulatory CD4+Foxp3+ T cells control memory responses to influenza virus infection. J Immunol. 2013;190(7):3438–46. 10.4049/jimmunol.1203140 23467933 PMC3608733

[21] Becher B, Waisman A, Lu LF. Conditional Gene-Targeting in Mice: Problems and Solutions. Immunity. 2018;48(5):835–6. 10.1016/j.immuni.2018.05.002 29768166 PMC7771386

[22] Penaloza-MacMaster P, Barber DL, Wherry EJ, Provine NM, Teigler JE, Parenteau L, et al Vaccine-elicited CD4 T cells induce immunopathology after chronic LCMV infection. Science. 2015;347(6219):278–82. 10.1126/science.aaa2148 25593185 PMC4382081

[23] Karauzum H, Haudenschild CC, Moore IN, Mahmoudieh M, Barber DL, Datta SK. Lethal CD4 T Cell Responses Induced by Vaccination Against Staphylococcus aureus Bacteremia. J Infect Dis. 2017;215(8):1231–9. 10.1093/infdis/jix096 28329242 PMC5853805

[24] Roman E, Miller E, Harmsen A, Wiley J, Von Andrian UH, Huston G, et al CD4 effector T cell subsets in the response to influenza: heterogeneity, migration, and function. J Exp Med. 2002;196(7):957–68. 10.1084/jem.20021052 12370257 PMC2194021

[25] Murphy KM, Heimberger AB, Loh DY. Induction by antigen of intrathymic apoptosis of CD4+CD8+TCRlo thymocytes in vivo. Science. 1990;250(4988):1720–3. 10.1126/science.2125367 2125367

[26] McKinstry KK, Strutt TM, Kuang Y, Brown DM, Sell S, Dutton RW, et al Memory CD4+ T cells protect against influenza through multiple synergizing mechanisms. J Clin Invest. 2012;122(8):2847–56. 10.1172/JCI63689 22820287 PMC3408751

[27] Swain SL, McKinstry KK, Strutt TM. Expanding roles for CD4(+) T cells in immunity to viruses. Nat Rev Immunol. 2012;12(2):136–48. 10.1038/nri3152 22266691 PMC3764486

[28] McKinstry KK, Strutt TM, Swain SL. Regulation of CD4+ T-cell contraction during pathogen challenge. Immunol Rev. 2010;236:110–24. 10.1111/j.1600-065X.2010.00921.x 20636812 PMC2908916

[29] Richards KA, Chaves FA, Sant AJ. The memory phase of the CD4 T-cell response to influenza virus infection maintains its diverse antigen specificity. Immunology. 2011;133(2):246–56. 10.1111/j.1365-2567.2011.03435.x 21517839 PMC3088986

[30] Caton AJ, Gerhard W. The diversity of the CD4+ T cell response in influenza. Semin Immunol. 1992;4(2):85–90. 1352154

[31] Strutt TM, McKinstry KK, Dibble JP, Winchell C, Kuang Y, Curtis JD, et al Memory CD4+ T cells induce innate responses independently of pathogen. Nat Med. 2010;16(5):558–64, 1p following 64. 10.1038/nm.2142 20436484 PMC2927232

[32] McKinstry KK, Strutt TM, Buck A, Curtis JD, Dibble JP, Huston G, et al IL-10 deficiency unleashes an influenza-specific Th17 response and enhances survival against high-dose challenge. J Immunol. 2009;182(12):7353–63. 10.4049/jimmunol.0900657 19494257 PMC2724021

[33] Sanders CJ, Vogel P, McClaren JL, Bajracharya R, Doherty PC, Thomas PG. Compromised respiratory function in lethal influenza infection is characterized by the depletion of type I alveolar epithelial cells beyond threshold levels. Am J Physiol Lung Cell Mol Physiol. 2013;304(7):L481–8. 10.1152/ajplung.00343.2012 23355384 PMC3627938

[34] Julander JG, Kesler K, Van Wettere AJ, Morrey JD, Smee DF. The use of plethysmography in determining the severity of lung pathology in a mouse model of minimally lethal influenza virus infection. Antiviral Res. 2014;108:10–3. 10.1016/j.antiviral.2014.05.002 24837607

[35] Welten SP, Redeker A, Franken KL, Benedict CA, Yagita H, Wensveen FM, et al CD27-CD70 costimulation controls T cell immunity during acute and persistent cytomegalovirus infection. J Virol. 2013;87(12):6851–65. 10.1128/JVI.03305-12 23576505 PMC3676092

[36] Peperzak V, Xiao Y, Veraar EA, Borst J. CD27 sustains survival of CTLs in virus-infected nonlymphoid tissue in mice by inducing autocrine IL-2 production. J Clin Invest. 2010;120(1):168–78. 10.1172/JCI40178 19955658 PMC2798690

[37] Perrone LA, Szretter KJ, Katz JM, Mizgerd JP, Tumpey TM. Mice lacking both TNF and IL-1 receptors exhibit reduced lung inflammation and delay in onset of death following infection with a highly virulent H5N1 virus. J Infect Dis. 2010;202(8):1161–70. 10.1086/656365 20815704 PMC2941567

[38] Schmitz N, Kurrer M, Bachmann MF, Kopf M. Interleukin-1 is responsible for acute lung immunopathology but increases survival of respiratory influenza virus infection. J Virol. 2005;79(10):6441–8. 10.1128/JVI.79.10.6441-6448.2005 15858027 PMC1091664

[39] Svitek N, Rudd PA, Obojes K, Pillet S, von Messling V. Severe seasonal influenza in ferrets correlates with reduced interferon and increased IL-6 induction. Virology. 2008;376(1):53–9. 10.1016/j.virol.2008.02.035 18420248

[40] Crowe CR, Chen K, Pociask DA, Alcorn JF, Krivich C, Enelow RI, et al Critical role of IL-17RA in immunopathology of influenza infection. J Immunol. 2009;183(8):5301–10. 10.4049/jimmunol.0900995 19783685 PMC3638739

[41] Munitic I, Kuka M, Allam A, Scoville JP, Ashwell JD. CD70 deficiency impairs effector CD8 T cell generation and viral clearance but is dispensable for the recall response to lymphocytic choriomeningitis virus. J Immunol. 2013;190(3):1169–79. 10.4049/jimmunol.1202353 23269247 PMC3552005

[42] Hamada H, Bassity E, Flies A, Strutt TM, Garcia-Hernandez Mde L, McKinstry KK, et al Multiple redundant effector mechanisms of CD8+ T cells protect against influenza infection. J Immunol. 2013;190(1):296–306. 10.4049/jimmunol.1200571 23197262 PMC3864858

[43] Spolski R, Li P, Leonard WJ. Biology and regulation of IL-2: from molecular mechanisms to human therapy. Nat Rev Immunol. 2018;18(10):648–59. 10.1038/s41577-018-0046-y 30089912

[44] Mier JW, Vachino G, van der Meer JW, Numerof RP, Adams S, Cannon JG, et al Induction of circulating tumor necrosis factor (TNF alpha) as the mechanism for the febrile response to interleukin-2 (IL-2) in cancer patients. J Clin Immunol. 1988;8(6):426–36. 3265420 10.1007/BF00916947

[45] Boyman O, Kovar M, Rubinstein MP, Surh CD, Sprent J. Selective stimulation of T cell subsets with antibody-cytokine immune complexes. Science. 2006;311(5769):1924–7. 10.1126/science.1122927 16484453

[46] Krieg C, Letourneau S, Pantaleo G, Boyman O. Improved IL-2 immunotherapy by selective stimulation of IL-2 receptors on lymphocytes and endothelial cells. Proc Natl Acad Sci U S A. 2010;107(26):11906–11. 10.1073/pnas.1002569107 20547866 PMC2900642

[47] Boyman O, Sprent J. The role of interleukin-2 during homeostasis and activation of the immune system. Nat Rev Immunol. 2012;12(3):180–90. 10.1038/nri3156 22343569

[48] Rosenstein M, Ettinghausen SE, Rosenberg SA. Extravasation of intravascular fluid mediated by the systemic administration of recombinant interleukin 2. J Immunol. 1986;137(5):1735–42. 3528289

[49] Shi C, Pamer EG. Monocyte recruitment during infection and inflammation. Nat Rev Immunol. 2011;11(11):762–74. 10.1038/nri3070 21984070 PMC3947780

[50] Assier E, Jullien V, Lefort J, Moreau JL, Di Santo JP, Vargaftig BB, et al NK cells and polymorphonuclear neutrophils are both critical for IL-2-induced pulmonary vascular leak syndrome. J Immunol. 2004;172(12):7661–8. 10.4049/jimmunol.172.12.7661 15187148

[51] Ettinghausen SE, Puri RK, Rosenberg SA. Increased vascular permeability in organs mediated by the systemic administration of lymphokine-activated killer cells and recombinant interleukin-2 in mice. J Natl Cancer Inst. 1988;80(3):177–88. 10.1093/jnci/80.3.177 3258039

[52] Gabrielli S, Ortolani C, Del Zotto G, Luchetti F, Canonico B, Buccella F, et al The Memories of NK Cells: Innate-Adaptive Immune Intrinsic Crosstalk. J Immunol Res. 2016;2016:1376595 10.1155/2016/1376595 28078307 PMC5204097

[53] Hoegl S, Ehrentraut H, Brodsky KS, Victorino F, Golden-Mason L, Eltzschig HK, et al NK cells regulate CXCR2+ neutrophil recruitment during acute lung injury. J Leukoc Biol. 2017;101(2):471–80. 10.1189/jlb.3A0516-227R 27601626 PMC5235908

[54] Waggoner SN, Cornberg M, Selin LK, Welsh RM. Natural killer cells act as rheostats modulating antiviral T cells. Nature. 2012;481(7381):394–8. 10.1038/nature10624 PMC3539796 22101430

[55] Carlin LE, Hemann EA, Zacharias ZR, Heusel JW, Legge KL. Natural Killer Cell Recruitment to the Lung During Influenza A Virus Infection Is Dependent on CXCR3, CCR5, and Virus Exposure Dose. Front Immunol. 2018;9:781 10.3389/fimmu.2018.00781 29719539 PMC5913326

[56] Camp JV, Jonsson CB. A Role for Neutrophils in Viral Respiratory Disease. Front Immunol. 2017;8:550. 28553293 10.3389/fimmu.2017.00550 PMC5427094

[57] Darrah PA, Patel DT, De Luca PM, Lindsay RW, Davey DF, Flynn BJ, et al Multifunctional TH1 cells define a correlate of vaccine-mediated protection against Leishmania major. Nat Med. 2007;13(7):843–50. 10.1038/nm1592 17558415

[58] Burel JG, Apte SH, Groves PL, McCarthy JS, Doolan DL. Polyfunctional and IFN-gamma monofunctional human CD4+ T cell populations are molecularly distinct. JCI Insight. 2017;2(3):e87499 10.1172/jci.insight.87499 28194431 PMC5291737

[59] DiPiazza A, Laniewski N, Rattan A, Topham DJ, Miller J, Sant AJ. CD4 T Cell Epitope Specificity and Cytokine Potential Are Preserved as Cells Transition from the Lung Vasculature to Lung Tissue following Influenza Virus Infection. J Virol. 2018;92(13). 10.1128/JVI.00377-18 PMC6002724 29669836

[60] Rogers PR, Croft M. Peptide dose, affinity, and time of differentiation can contribute to the Th1/Th2 cytokine balance. J Immunol. 1999;163(3):1205–13. 10415015

[61] Strutt TM, McKinstry KK, Marshall NB, Vong AM, Dutton RW, Swain SL. Multipronged CD4(+) T-cell effector and memory responses cooperate to provide potent immunity against respiratory virus. Immunol Rev. 2013;255(1):149–64. 10.1111/imr.12088 23947353 PMC4206082

[62] Sant AJ, Richards KA, Nayak J. Distinct and complementary roles of CD4 T cells in protective immunity to influenza virus. Curr Opin Immunol. 2018;53:13–21. 10.1016/j.coi.2018.03.019 29621639 PMC6141328

[63] Zens KD, Farber DL. Memory CD4 T cells in influenza. Curr Top Microbiol Immunol. 2015;386:399–421. 10.1007/82_2014_401 25005927 PMC4339101

[64] Lee WW, Teo TH, Lum FM, Andiappan AK, Amrun SN, Renia L, et al Virus infection drives IL-2 antibody complexes into pro-inflammatory agonists in mice. Sci Rep. 2016;6:37603 10.1038/srep37603 27886209 PMC5122839

[65] Ettinghausen SE, Lipford EH 3rd, Mule JJ, Rosenberg SA. Systemic administration of recombinant interleukin 2 stimulates in vivo lymphoid cell proliferation in tissues. J Immunol. 1985;135(2):1488–97. 3891854

[66] Califano D, Furuya Y, Roberts S, Avram D, McKenzie ANJ, Metzger DW. IFN-gamma increases susceptibility to influenza A infection through suppression of group II innate lymphoid cells. Mucosal Immunol. 2018;11(1):209–19. 10.1038/mi.2017.41 28513592 PMC5693789

[67] McKinstry KK, Strutt TM, Swain SL. The effector to memory transition of CD4 T cells. Immunol Res. 2008;40(2):114–27. 10.1007/s12026-007-8004-y 18213525

[68] Westerhof LM, McGuire K, MacLellan L, Flynn A, Gray JI, Thomas M, et al Multifunctional cytokine production reveals functional superiority of memory CD4 T cells. Eur J Immunol. 2019. 10.1002/eji.201848026 PMC6900100 31177549

[69] Arimilli S, Palmer EM, Alexander-Miller MA. Loss of function in virus-specific lung effector T cells is independent of infection. J Leukoc Biol. 2008;83(3):564–74. 10.1189/jlb.0407215 18079210 PMC11650726

[70] Carter L, Fouser LA, Jussif J, Fitz L, Deng B, Wood CR, et al PD-1:PD-L inhibitory pathway affects both CD4(+) and CD8(+) T cells and is overcome by IL-2. Eur J Immunol. 2002;32(3):634–43. 10.1002/1521-4141(200203)32:3<634::AID-IMMU634>3.0.CO;2-9 11857337

[71] Wei F, Zhong S, Ma Z, Kong H, Medvec A, Ahmed R, et al Strength of PD-1 signaling differentially affects T-cell effector functions. Proc Natl Acad Sci U S A. 2013;110(27):E2480–9. 10.1073/pnas.1305394110 23610399 PMC3703988

[72] McNally B, Ye F, Willette M, Flano E. Local blockade of epithelial PDL-1 in the airways enhances T cell function and viral clearance during influenza virus infection. J Virol. 2013;87(23):12916–24. 10.1128/JVI.02423-13 24067957 PMC3838157

[73] Rutigliano JA, Sharma S, Morris MY, Oguin TH 3rd, McClaren JL, Doherty PC, et al Highly pathological influenza A virus infection is associated with augmented expression of PD-1 by functionally compromised virus-specific CD8+ T cells. J Virol. 2014;88(3):1636–51. 10.1128/JVI.02851-13 24257598 PMC3911576

[74] Sun J, Dodd H, Moser EK, Sharma R, Braciale TJ. CD4+ T cell help and innate-derived IL-27 induce Blimp-1-dependent IL-10 production by antiviral CTLs. Nat Immunol. 2011;12(4):327–34. 10.1038/ni.1996 21297642 PMC3079402

[75] Sun J, Madan R, Karp CL, Braciale TJ. Effector T cells control lung inflammation during acute influenza virus infection by producing IL-10. Nat Med. 2009;15(3):277–84. 10.1038/nm.1929 19234462 PMC2693210

[76] Porichis F, Hart MG, Massa A, Everett HL, Morou A, Richard J, et al Immune Checkpoint Blockade Restores HIV-Specific CD4 T Cell Help for NK Cells. J Immunol. 2018;201(3):971–81. 10.4049/jimmunol.1701551 29934472 PMC6064609

[77] Wherry EJ, Kurachi M. Molecular and cellular insights into T cell exhaustion. Nat Rev Immunol. 2015;15(8):486–99. 10.1038/nri3862 26205583 PMC4889009

[78] Garber K. Cytokine resurrection: engineered IL-2 ramps up immuno-oncology responses. Nat Biotechnol. 2018;36(5):378–9. 10.1038/nbt0518-378 29734296

[79] Thomas PG, Brown SA, Yue W, So J, Webby RJ, Doherty PC. An unexpected antibody response to an engineered influenza virus modifies CD8+ T cell responses. Proc Natl Acad Sci U S A. 2006;103(8):2764–9. 10.1073/pnas.0511185103 16473934 PMC1413843

